# The use of artificial nutrition at the end-of-life: a cross-sectional survey exploring the beliefs and decision-making among physicians and nurses

**DOI:** 10.1007/s00520-025-09310-2

**Published:** 2025-03-17

**Authors:** Christophe Pala, Claudia Gamondi, Steffen Eychmuller, Francois Herrmann, Sophie Pautex

**Affiliations:** 1 Department of Rehabilitation and Geriatrics, Division of Palliative Medicine, Geneva University Hospitals, 11 Chemin de La Savonnière, 1245 Collonge-Bellerive, Geneve, Switzerland; 2 Palliative and Supportive Care Service, Lausanne University Hospital and University of Lausanne, Lausanne, Switzerland; 3 University Centre for Palliative Care, Inselspital, University Hospital Bern, Bern, Switzerland; 4 Department of Rehabilitation and Geriatrics, Division of Geriatrics, Geneva University Hospitals and Faculty of Medicine, Geneva, Switzerland; 5 Department of Rehabilitation and Geriatrics, Division of Palliative Medicine, Geneva University Hospitals and Faculty of Medicine, Geneve, Switzerland

**Keywords:** End-of-life, Terminal care, Palliative care, Artificial feeding/nutrition, Attitude of health personnel, Health personnel knowledge

## Abstract

**Background:**

The use of artificial nutrition in the last month of life raises many concerns for patients, relatives, and healthcare professionals.

**Aim:**

To describe physicians and nurses’ beliefs, knowledge, and decision-making related to introducing and withdrawing artificial nutrition at the end-of-life. Physicians and nurses’ factors affecting these decisions were examined.

**Design:**

A cross-sectional study was conducted between May and July 2022. A questionnaire was sent by email to physicians and nurses.

**Setting and participants:**

Physicians and nurses working in internal medicine, oncology, and palliative medicine divisions in three Swiss University Hospitals.

**Results:**

Two hundred and thirty physicians and nurses completed the survey (21% response rate). Most responders, aged 25–45, were women with < 10 years of experience, 61% lacked palliative care experience. End-of-life decision-making on artificial nutrition was reported as common by 89%. Whereas physicians and nurses played an important role in the decision, fulfilling patients’ wishes (84% of cases) tended to dominate over professionals’ intentions (physicians 52%, nurses 67%) as motivators at final decision. The main reasons for introducing artificial nutrition included improving nutritional status (54%), reducing broncho-aspiration (67%), and preventing pressure ulcers (53%). Having palliative care experience was the only variable modifying the beliefs of these motivations.

**Conclusion:**

Whereas decisions on artificial nutrition at the end of life are common they may be mostly guided by physicians and nurses’ beliefs, and patients’ requests more than by robust evidence. Fostering palliative care education is pivotal. Our results emphasize the need to improve physicians and nurses’ awareness of the complex interplay between values and evidence when decisions concerning artificial nutrition are taken.

**Supplementary Information:**

The online version contains supplementary material available at 10.1007/s00520-025-09310-2.

## What is already known about the topic

• The decision to withdraw/withhold artificial nutrition at the end of life remains controversial, even if frequent.

 • Artificial nutrition may have limited benefits for patients at the end of life. It may not necessarily improve quality of life or survival and can be associated with risks and burdens, including complications such as aspiration, infection, and discomfort.

## What this paper adds

 • The decision concerning artificial nutrition at the end of life remains influenced by the patient's wishes and physicians and nurses' beliefs more than by evidence. Physicians and nurses can hold misleading information concerning artificial nutrition's possibilities improving nutritional status, reducing broncho-aspiration, and preventing bedsores at the end of life.

 • Having a palliative care experience might positively influence adherence to recommendations concerning the use of artificial nutrition at the end of life.

 • Improving physicians and nurses' awareness about the impact on the decision-making of their values concerning the significance of food at the end of life might enhance adherence to clinical recommendations.

## Implications for practice, theory, or policy

 • An interprofessional approach is needed to make decisions about artificial nutrition at the end of life, ensuring that the patient's autonomy is respected but also taking into account the principles of beneficence and non-maleficence in order to provide ethically and clinically appropriate care. Promoting physicians and nurses' education in palliative care and advanced planning with specific attention to artificial nutrition at the of life my reduce bias in decision-making concerning artificial nutrition at the end of life. 

## Introduction

At the end of life, nutrition raises many issues for patients, their relatives, and healthcare professionals. The Larousse dictionary defines the end of life as “the last months, weeks of days of a person suffering from a progressive and incurable disease.” The literary definition remains vague, as does the absence of a universal scientific definition of this concept often referred to as “end of life, terminal ill, actively dying.” In many western cultures, nutrition represents life and its withdrawal may be perceived to hasten death [1]. Several expert recommendations have been published regarding the indications for artificial nutrition in medicine, specifically at the end-of-life [2–8]. Nevertheless, the implementation of these recommendations shows significant variability, influenced by factors such as the patient’s individual circumstances, the specific healthcare setting, and cultural considerations. This variability is partly attributable to the ongoing requirement for additional evidence and the diverse perspectives represented among healthcare professionals. For example, Groenwoud et al. [9] observed that 5% of physicians were against discontinuing artificial nutrition at the end of life because they believed it would reduce life expectancy by more than one month. Buiting et al. [10] showed that 35% of physicians thought nutrition withdrawal would reduce life expectancy by < 1 week and 4% by 1–4 weeks. Heuberger et al. [11] observed that physicians and nurses, regardless of their years of experience, thought they could reduce the occurrence of broncho-aspiration or improve the nutritional status of patients at the end of life despite the absence of recommendations supporting these affirmations [11].

Switzerland’s distinctiveness stems from its diversity (https://www.bfs.admin.ch/bfs/fr/home/statistiken/bevoelkerung/sprachen-religionen/religionen.html), situated at the intersection of multiple European countries. Additionally, it is a multilingual nation, with four official languages and cultures German, French, Italian, and Romansh. Therefore, we aimed to describe the attitudes, beliefs, and decision-making processes regarding artificial nutrition at the end-of-life of healthcare professionals working in the internal medicine, oncology, and palliative care divisions of university hospitals of three Switzerland’s different linguistic regions. We also aimed to analyze the influence of factors related to physicians and nurses and patients on attitudes, beliefs, and decision-making processes.

## Methods

### Study design

A cross-sectional survey was conducted between may and july 2022. The consensus-based checklist STROBE for reporting survey studies was followed to ensure comprehensive reporting and adherence to standards for survey studies.

### Sample characteristics

Swiss healthcare physicians and nurses working in the internal medicine, oncology, and palliative medicine divisions of the University Hospitals of Geneva (French region), University Hospital of Bern (InselSpital, IS) (German region), and Cantonal Hospital of Ticino (EOC) (Italian region) were included. A total of 1059 questionnaires were sent to all physicians and nurses working in the nine divisions (physicians: 493 (University Hospitals of Geneva 184; InselSpital 104; Cantonal Hospital of Ticino 205); nurses: 566 (University Hospitals of Geneva 246; InselSpital 110; Cantonal Hospital of Ticino 210)).

### Data collection

A thorough examination of existing literature was undertaken to develop the survey. The following keywords were used: end-of-life, terminal care, palliative care, patient care team, enteral feeding/nutrition, parenteral feeding/nutrition, artificial feeding/nutrition, nutritional support, attitude of health personnel, and health personnel knowledge. The search was conducted, including literature published between 1980 and 2022 [9–34].

The survey included a clinical vignette to establish the context, an introduction with definitions and 45 questions divided into 11 questions on physicians and nurses’ characteristics, nine on beliefs and 25 on the decision-making about artificial nutrition at the end of life. For the purpose the study we retained as definition of end of life, 1 month according to the data relating to the Palliative Performance Scale and the prognosis estimate according to the review accomplish by Baik et al. [35]. It contained mixed multiple-choice and free-text questions (see Appendix). The first author developed the survey in French and then validated it by the researchers’ team. During the pre-test phase of the questionnaire, we evaluated it using a “face validation” approach, ensuring that the objectives of the tool were clear and selecting a panel of evaluators representative of the target groups. The evaluators assessed the clarity of the items, their comprehensibility and their direct link with the objectives being studied. However, we did not measure reliability.

The questionnaire was tested with a panel of five physicians and five nurses and modified based on their feedback to improve understanding of the questions. Additionally, a definition of key concepts, such as “end-of-life” which was defined as within one month, was added.

Two native-speaking healthcare professionals finally translated the questionnaire into German and Italian. They made a back-forward translation to reinforce the validity of the translation.

### Survey administration

The online survey link was e-mailed to professionals using Microsoft Forms ®. A reminder was sent at three weeks.

After completing the survey, the responses were automatically captured and stored in an Excel database. Personal identifiers, such as names and email addresses, were not collected nor stored to ensure anonymity.

### Statistical analysis

The survey data were thoroughly cleaned to ensure data quality and integrity. This process involved checking for missing values and inconsistencies. Only complete or valid responses were included in the analysis. The data were then prepared for analysis by assigning numerical codes or categories to multiple-choice answers. We conducted a descriptive analysis of the frequencies of responses and percentages for each question. According to the variables distribution, we used Fisher’s exact tests to compare proportion and *t*-tests to compare continuous variables such as age or the number of years of experience between two group. According to the variables distribution, we used Mann–Whitney *U*-test to compare proportion and one-way analysis of variance to compare continuous variables such as age or the number of years of experience between three groups.

Bivariate linear regression models were used to determine the association between the answers to different questions while adjusting for language on the number of years of experience in health care (the dependant variable).

Bivariate logistic regression models were used to determine the association between the answers to different questions while adjusting for language on different binary variables such as profession (nurses versus physicians) and healthcare professionals’ opinions regarding the use of artificial nutrition at the end-of-life (no versus yes). The results obtained having been consistent with the other analyses and without a language effect, we have not reproduced these data.

All statistics were computed with STATA software version 17.0 to determine an association between the answer to the survey and some characteristics of the physicians and nurses: (1) years of experience; (2) experience in palliative care; (3) profession (physician/nurse); (4) previous professional experience of artificial nutrition in end-of-life; (5) personally favorable to artificial nutrition use in end-of-life; (6) favorable to artificial nutrition use even if loss of decision-making capacity; and (7) linguistic region. We carried out a linear regression analysis to identify possible regional differences between the three linguistic regions. The central question was to determine whether the number of years of experience influenced, according to these linguistic regions, perceptions of the effects of artificial nutrition. These perceptions included its potential impact on: improving nutritional status, reducing the risk of broncho-aspiration, preventing pressure sores, preventing the feeling of thirst, preventing the feeling of hunger, improving of asthenia, improvement of autonomy, reduction of pain, slowing of oncological progression, prolongation of life, improvement of quality of life, and perception of therapeutic relentlessness. Our analyses revealed no significant differences between the three linguistic regions regarding these variables.

#### Ethical considerations and financing

This study obtained ethical approval from the institutional review board (*Conseil d’évaluation des études ne relevant pas de la commission d’éthique de la recherche (CEENCER)*) and was conduct according to the Swiss legal requirements and the current version of the World Medical Association Declaration of Helsinki. Participant’s informed consent was obtained within the electronic survey before completing the questionnaire.

## Results

### Participants

Two-hundred thirty of one thousand fifty-nine physicians and nurses completed the questionnaire (21.7%) (University Hospitals of Geneva 21.4%; InselSpital 37.8%; Cantonal Hospital of Ticino 13.7%) after the recall and none of the 230 responses were excluded. Of them, 49.6% were physicians, and 50.4% were nurses (Table 1).

**Table 1:** Characteristics of physicians and nurses

*N* (%)	University Hospitals of Geneva French *N* = 92	InselSpital German *N* = 81	Cantonal Hospital of Ticino Italian *N* = 57	Total *N* = 230
Age (years)				
20–24	3 (3.26%)	10 (12%)	0 (0%)	13 (5.7%)
25–45	71 (77.17%)	54 (68%)	37 (64.91%)	162 (70.4%)
56–60	18 (19.56%)	10 (12%)	18 (31.58%)	46 (20%)
> 60	0 (0%)	2 (2%)	2 (3.51%)	4 (1.7%)
Sex				
Women	68 (78%)	60 (75%)	32 (56%)	160 (69.6%)
Men	24 (26%)	21 (26%)	25 (44%)	70 (30.4%)
Profession				
Physician	42 (46%)	39 (48%)	33 (58%)	114 (49.6%)
Nurses	50 (54%)	42 (52%)	24 (42%)	116 (50.4%)
Work experience (years)				
< 5	22 (23.92%)	21 (26%)	6 (10.53%)	49 (21.3%)
5–10	25 (27.17%)	32 (39%)	17 (29.82%)	74 (32.2%)
> 10	45 (48.91%)	28 (35%)	34 (59.65%)	107 (46.5%)
Palliative care experience				
Yes	30 (33%)	30 (37%)	30 (53%)	90 (39.1%)
No	62 (67%)	51 (63%)	27 (47%)	140 (60.9%)
Experience with discontinuing artificial nutrition				
Yes	83 (90%)	51 (62%)	49 (86%)	205 (89.1%)
No	9 (10%)	30 (38%)	8 (14%)	25 (10.9%)
Religion				
Christian	43 (46.7%)	53 (68%)	33 (57.89%)	129 (56%)
Jewish	1 (1.1%)	0 (0%)	0 (0%)	1 (0.4%)
Islam	4 (4.3%)	1 (1.3%)	0 (0%)	5 (2.2%)
Hinduism	1 (1.11%)	1 (1.3%)	0 (0%)	2 (0.5%)
No	35 (38%)	21 (27%)	20 (35.1%)	76 (33%)
No answer	8 (8.7%)	1 (1.3%)	4 (7.02%)	13 (5.7%)

### Characteristics of physicians and nurses (Table 1)

Women made up the majority of the sample, with 70% of respondents. In terms of age distribution, 70% of participants were between 25 and 45 years old. In terms of professional experience, 53% had less than 10 years’ experience, while 32.2% had between 5 and 10 years’ experience and only 21% had less than 5 years’ experience. The Cantonal Hospital in Ticino had the highest proportion of staff with more than 10 years’ experience (59.7%), compared with 48.9% at the University Hospitals in Geneva and 35% at the InselSpital. Conversely, junior doctors and nurses were more prevalent in the latter two hospitals (23.9% and 26% respectively) than in Ticino (only 10.5%). We can hypothesize that institutional, demographic, and staffing differences could explain this.

Regarding regional affiliation, we found that the majority of participants declared themselves to be Christian (56%), although there were regional differences: 65% at the InselSpital, 57.9% at the Cantonal Hospital of Ticino and 46.7% at the university hospital in Geneva. These differences are part of a different cultural context between these 3 linguistic regions of Switzerland. Around a third (33%) of doctors and nurses reported no religion, and this was more marked at Geneva University Hospital (38%). Other religions, such as Islam, Judaism, and Hinduism, were poorly represented in the group studied (< 3%), and 5.7% refused to answer this question. These figures are consistent with those of the Swiss Federal Office of Public Health [36] and could explain this. Finally, 89% of the participants had already experienced a decision to discontinue artificial nutrition at the end of life. Our linear regression analyses revealed no significant difference among the three groups from different linguistic regions, as we mentioned before in the method.

### Beliefs toward artificial nutrition at the end of life (Table 2)

**Table 2:** Beliefs of physicians and nurses toward artificial nutrition

*N* (%)	University Hospitals of Geneva French *N* = 92	InselSpital German *N* = 81	Cantonal Hospital of Ticino Italian *N* = 57	Total *N* = 230
In favor of artificial nutrition use in end-of-life				
Yes	21 (23%)	14 (17%)	4 (16%)	39 (17%)
No	71 (77%)	67 (83%)	53 (84%)	191 (83%)
In favor of artificial nutrition use in case of loss of capacity				
Yes	15 (16%)	10 (12%)	5 (9%)	30 (13%)
No	77 (84%)	71 (88%)	52 (91%)	200 (87%)

The majority of physicians and nurses opposed the implementation of artificial nutrition in end-of-life situations. Despite a higher number of physicians and nurses from the French region supporting its use, most were against it when dealing with a loss of mental capacity.

### Beliefs of physicians and nurses associated with artificial nutrition at the end-of-life.

Various opinions related to the introduction of artificial nutrition in end-of-life patients were identified (Table 3). Regarding initiation, 54.3% of physicians and nurses believe that it could improve nutritional status and this proportion is highest in the University Hospital of Geneva with 66.3% compared to InselSpital (46% and Hôpital Cantonal du Ticino) (47.3%). Prevention of broncho-aspiration events is thought of by 67% of participants, with a greater proportion at the InselSpital (73%) than at the University Hospitals of Geneva (62%) and Cantonal Hospital of Ticino (66.7%).

**Table 3:** Beliefs related to the initiation/cessation/approach as a medical therapy of artificial nutrition at the end of life, categorized by regions

*N* (%)	University Hospitals of Geneva French *N* = 92	InselSpital German *N* = 81	Cantonal Hospital of Ticino Italian *N* = 57		Total *N* = 230
Beliefs associated to the introduction of artificial nutrition in end-of-life					
Improvement of nutritional status					
Yes	61 (66.3%)	37 (46%)		27 (47.3%)	125 (54.3%)
No	31 (33.7%)	44 (54%)		30 (52.6%)	105 (45.7%)
Broncho-aspiration prevention					
Yes	57 (62%)	59 (73%)		38 (66.7%)	154 (67%)
No	35 (38%)	22 (27%)		19 (33.3%)	76 (33%)
Prevention of bedsores					
Yes	68 (73.9%)	24 (30%)		29 (51%)	121 (53%)
No	24 (26.1%)	57 (70%)		28 (49%)	109 (47%)
Thirst prevention					
Yes	25 (27.2%)	16 (20%)		16 (28.1%)	57 (25%)
No	67 (72.8%)	65 (80%)		41 (71.9%)	173 (75%)
Hunger prevention					
Yes	40 (43.5%)	36 (44.4%)		21 (36.8%)	97 (42%)
No	52 (56.5%)	45 (55.6%)		36 (63.2%)	133 (58%)
Improvement asthenia					
Yes	47 (51.1%)	32 (40%)		24 (42.1%)	103 (45%)
No	45 (48.9%)	49 (60%)		33 (57.9%)	127 (55%)
Improvement in daily activity					
Yes	20 (21.7%)	16 (20%)		6 (10.5%)	42 (18%)
No	72 (78.3%)	65 (80%)		51 (89.5%)	188 (82%)
Decrease pain intensity					
Yes	9 (9.8%)	11 (14%)		4 (7%)	24 (10.4) %
No	83 (90.2%)	70 (86%)		53 (93%)	206 (89.6) %
Progression of cancer					
Yes	4 (4.3%)	8 (10%)		2 (3.5%)	14 (6%)
No	88 (95.7%)	73 (90%)		55 (96.5%)	216 (94%)
Prolonging life					
Yes	36 (39.1%)	35 (43%)		16 (28.1%)	87 (38%)
No	56 (60.9%)	46 (57%)		41 (71.9%)	143 (62%)
Improvement in quality of life					
Yes	44 (47.8%)	36 (44%)		8 (14%)	88 (38%)
No	48 (52.2%)	45 (56%)		49(86%)	142(62%)
Beliefs associated to the withdrawal of artificial nutrition in end-of-life					
Neglect Yes	5 (5.4%)	4 (5%)		9 (15.8%)	18 (7.8%)
No	87 (94.6%)	77 (95%)		48 (84.2%)	212 (92.2%)
A form of assisted suicide					
Yes	3 (3.3%)	3 (4%)		2 (3.5%)	8 (3.5%)
No	89 (96.7%)	78 (96%)		55 (96.5%)	222 (96.5%)
A form of euthanasia					
Yes	1 (1.1%)	5 (6%)		1 (1.8%)	7 (3%)
No	91 (98.9%)	76 (94%)		56 (98.2%)	223 (97%)
Comfort					
Yes	36 (39%)	42 (52%)		37 (65%)	115 (50%)
No	56 (61%)	39 (48%)		20 (35%)	115 (50%)
Beliefs associated to artificial nutrition as medical therapy in end-of-life					
Aggressive treatment					
Yes	58 (63%)	66 (81%)		46 (80.7%)	170 (73.9%)
No	34 (37%)	15 (19%)		11 (19.3%)	60 (26.1%)
Palliative treatment					
Yes	43 (47%)	28 (35%)		20 (35%)	91 (40%)
No	49 (53%)	53 (65%)		37 (65%)	139 (60%)
A treatment					
Yes	66 (72%)	74 (91%)		47 (82%)	187 (81%)
No	26 (28%)	7 (9%)		10 (18%)	43 (19%)

Beliefs regarding the prevention of pressure ulcers were more frequent in the University Hospitals of Geneva (73.9%) than in the other two. The effect on the feeling of thirst or hunger was not generally recognized in the 3 hospitals. In addition, only 18% of participants thought that artificial nutrition improves daily quality and only 10.4% imagine a potential impact on reducing the intensity of pain. Concerning withdrawal, this act was associated with neglect for 7.8% of participants and this belief was more frequent in the Cantonal Hospital of Ticino (15.8%). Only 3.5% of doctors and nurses associated withdrawal with assisted suicide and 3% with euthanasia.

The possibility of artificial nutrition contributing to patient comfort is mentioned mainly among the participants with a predominance at the Cantonal Hospital of Ticino (65%) compared to the InselSpital (52%) and University Hospitals of Geneva with only 39%

The classification of artificial nutrition as a medical treatment also varies. It is perceived as an aggressive intervention by 73.9% of respondents, with this view being more prevalent in InselSpital (81%) and Cantonal Hospital of Ticino (80.7%) than in University Hospitals of Geneva (63%). Only 40% consider it part of a palliative approach, with lower recognition in InselSpital and the Cantonal Hospital of Ticino (35%) compared to University Hospitals of Geneva (47%). Finally, 81% of participants identify artificial nutrition as a medical treatment, with the highest proportion in InselSpital (91%).

### Decision-making process associated with artificial nutrition at end-of-life (Table 4)

**Table 4:** Decision-making process associated with artificial nutrition at end-of-life

*N* (%)	University Hospitals of Geneva French *N* = 92		InselSpital German *N* = 81	Cantonal Hospital of Ticino Italian *N* = 57	Total *N* = 230
Introduction/withdrawal of artificial nutrition is an interprofessional decision					
Yes		84 (91%)	78 (96%)	47 (82%)	209 (91%)
No		8 (91%)	3 (4%)	10 (18%)	21 (9%)
Members of the interprofessional team included in the decision					
Physicians					
Yes		88 (95.7%)	80 (99%)	56 (98%)	224 (97.4%)
No		4 (4.3%)	1 (1%)	1 (2%)	6 (2.6%)
Nurses					
Yes		88 (95.7%)	77 (95%)	55 (96.5%)	220 (96%)
No		4 (4.3%)	4 (5%)	2 (3.5%)	10 (4%)
Nursing assistant					
Yes		62 (67.4%)	41 (51%)	49 (86%)	152 (66%)
No		30 (32.6%)	40 (49%)	8 (14%)	78 (34%)
Others					
Yes		76 (82.6%)	51 (63%)	43 (75.4%)	170 (74%)
No		16 (17.4%)	30 (37%)	14 (24.6%)	60 (26%)
In the event that the patient is unable to participate in the decision-making process, the individuals encompassed are:					
Relatives					
Yes		91 (99%)	78 (96%)	53 (93%)	222 (96%)
No		1 (1%)	3 (4%)	4 (7%)	8 (4%)
Interprofessional team					
Yes		86 (94%)	68 (84%)	54 (95%)	208 (90%)
No		6 (6%)	13 (16%)	3 (5%)	22 (10%)
General practitioner					
Yes		62 (67%)	33 (41%)	39 (68%)	134 (58%)
No		30 (33%)	48 (59%)	18 (32%)	96 (42%)
Others					
Yes		1 (1%)	3 (4%)	0 (0%)	4 (2%)
No		91 (99%)	78 (96%)	57 (100%)	226 (98%)
Decision-making criteria to start or to withdraw artificial nutrition in end-of-life					
Life expectancy					
Yes		76 (82.6%)	73 (90%)	54 (94.7%)	203 (88%)
No		16 (17.4%)	8 (10%)	3 (5.3%)	27 (12%)
Quality of life					
Yes		91 (98.9%)	81 (100%)	57 (100%)	229 (99%)
No		1 (1.1%)	0 (0%)	0 (0%)	1 (1%)
Nutritional status					
Yes		69(75%)	55 (68%)	35 (61.4%)	159 (69%)
No		23 (25%)	26 (32%)	22 (38.6%)	71 (31%)
Age					
Yes		30 (32.6%)	22 (27%)	17 (29.8%)	69 (30%)
No		62 (67.4%)	59 (73%)	40 (70.2%)	161 (70%)
Comorbidities					
Yes		74 (80.4%)	64 (79%)	43 (75.4%)	181 (79%)
No		18 (19.6%)	17 (21%)	14 (24.6%)	49 (21%)
Group’s opinion					
Yes		19 (20.7%)	14 (17%)	13 (22.8%)	46 (20%)
No		73 (79.3%)	67 (83%)	44 (77.2%)	184 (80%)
Justice Yes No		31 (34%) 61 (66%)	20 (25%) 61 (75%)	22 (39%) 35 (61%)	73 (32%) 157(68%)
Bienfaisance Yes No		81 (88%) 11 (12%)	78 (96%) 3 (4%)	48 (84%) 9 (16%)	207 (90%) 23 (10%)
Non-maleficence Yes No		80 (87%) 12 (13%)	59 (73%) 22 (27%)	55 (96.5%) 2 (3%)	194 (84%) 36 (16%)
Autonomy Yes No		57 (62%) 35 (38%)	74 (91%) 7 (9%)	45 (79%) 12 (21%)	176 (77%) 54 (23%)

Most participants identified the introduction or withdrawal of artificial nutrition as an interprofessional decision (Table 4). However, in the Italian region, physicians reported sharing their decision less frequently (82%) than in other regions (96% InselSpital and 91% University Hospitals of Geneva). This suggests differences in approach, possibly cultural or organizational, in the integration of different professionals. In instances of impaired mental capacity, the involvement of the general practitioner was predominantly desired by caregivers in the regions of Geneva and Ticino but was in the minority at InselSpital. Regarding ethical principles influencing the decision-making process, no regional differences were observed. The principles of beneficence (*N* = 207/230, 90%), non-maleficence (*N* = 184/230, 84%) and autonomy (*N* = 176/230, 77%) were the most frequent ethical justifications to stop artificial nutrition. The key factors influencing initiation or withdrawal of artificial nutrition included life expectancy, quality of life, nutritional status, and comorbidities. The patient’s age was not a major decision-making factor (Table 4).

Others: other members of interprofessional team (e.g., physiotherapists, occupational therapists, psychologist, dieticians …).

The majority of physicians and nurses expressed the need for interprofessional support when deciding to discontinue artificial nutrition, 98% of physicians (*N* = 226/230), 92% of nurses (*N* = 211/230) and 78& of dieticians (*N* = 180/230).

### Association between physicians and nurses’ characteristics, beliefs, and decision-making processes related to introducing artificial nutrition in end-of-life (Table 5)

**Table 5:** Beliefs associated with the introduction of artificial nutrition at end-of-life according to palliative care experience

No experience in palliative care *N* = 140		Experience in palliative care *N* = 90	*P* Value
Improvement in nutritional status			
Yes	85 (60.7%)	40 (44.4%)	0.021
No	55 (39.3%)	50 (55.6%)	
Prevention of broncho-aspiration			
Yes	103 (73.6%)	52 (57.8%)	0.014
No	37 (26.4%)	38 (42.2%)	
Prevention of bedsores			
Yes	82 (58.6%)	39 (43.3%)	0.030
No	58 (41.4%)	51 (56.7%)	
Improvement of asthenia			
Yes	73 (52.1%)	27 (30.0%)	0.001
No	67 (47.9%)	63 (70%)	
Improvement of dependency			
Yes	31 (22.1%)	10 (11.1%)	0.035
No	109 (77.9%)	80 (88.9%)	
Improvement quality of life			
Yes	68 (48.6%)	19 (21.1%)	< 0.005
No	72 (51.4%)	71 (78.9%)	
Support patient’s decision to continue artificial nutrition at end of life			
Yes	132 (94.3%)	77 (85.6%)	0.034
No	8 (5.7%)	13 (14.4%)	

The duration of physicians and nurses’ work experience not significantly associated with their views regarding artificial nutrition, including indications, roles, ethical challenges, or patient comfort at end-of-life. Similar professional backgrounds did not sway decision-making on initiating or ceasing artificial nutrition. Nevertheless, accumulated work experience in palliative care increase, a greater number of individuals considered the perpetuation of artificial nutrition at the end of life to be a hindrance to patient comfort.

Among physicians and nurses with palliative care experience, 19 out of 90 (21.1%) believed that artificial nutrition at the end of life could improve quality of life. In contrast, 68 out of 140 (48.6%) of those without such experience shared this belief (*p* < 0.005). Additionally, 94.3% of physicians and nurses (*N* = 132/140) without palliative care experience supported patients’ decision to continue artificial nutrition compared to 85.6% of those with palliative care experience (*N* = 77/90, *p* = 0.03).

Physicians and nurses expressed different beliefs. Nurses expressed a higher frequency of agreement than physicians regarding the preventive effects of artificial nutrition on thirst (49.6% vs 22.9%, *p* = 0.02) and its potential to prolong life (44.3% vs 30.4%, *p* = 0.04). On the other hand, physicians more regularly considered variables such as life expectancy (95.7% vs 80.9%, *p* = 0.0007) and nutritional status (77.4% vs 61.7%, *p* = 0.014) in their decision-making process. Additionally, nurses were more inclined to seek advice from the ethics committee (*N* = 54/115, 46.9%) compared to physicians (*N* = 37/115,32.2% *p* = 0.03).

Active participation in decisions regarding the utilization of artificial nutrition at the end of life significantly shaped perceptions related to hunger prevention (39.5% vs 60%, *p* = 0.05) and the progression of cancer (4.4% vs 16%, *p* = 0.039). The majority of healthcare professionals (*N* = 190/205, 92.7%) who had prior experience in withdrawing artificial nutrition considered the patient’s quality of life a crucial factor, and this perspective was more prevalent than in the group without such experience (*N* = 20/25, 80%, *p* = 0.03).

In situations where a physician and nurse stated to personally believe in the benefits of artificial nutrition, their convictions were positively influencing their perceptions of its efficacy when offered to patients (Table 6).

**Table 6:** Beliefs associated with artificial nutrition according to the personal opinions of physicians and nurses

	Against the use artificial nutrition *N* = 186/230	Favorable to use artificial nutrition *N* = 44/230	*P* Value
Improvement of nutritional status			
Yes	93 (50.0%)	32 (72.7%)	0.007
No	93 (50%)	12 (27.3%)	
Prevention of broncho-aspiration			
Yes	115 (61.8%)	40 (90.9%)	< 0.005
No	71 (38.2%)	4 (9.1%)	
Prevention of bedsores			
Yes	88 (47.3%)	33 (75.0%)	0.001
No	98 (52.7%)	11 (25%)	
Prevention of thirst			
Yes	32 (17.2%)	25 (56.8%)	< 0.005
No	154 (82.8%)	19 (43.2%)	
Prevention of hunger			
Yes	61 (32.8%)	35 (79.5%)	< 0.005
No	125 (67.2%)	9 (20.5%)	
Improvement of asthenia			
Yes	69 (37.1%)	31 (70.5%)	< 0.005
No	117 (62.9%)	13 (29.5%)	
Improvement in activity of daily living			
Yes	21 (11.3%)	20 (45.5%)	< 0.005
No	165 (88.7%)	24 (54.5%)	
Decrease in pain intensity			
Yes	9 (4.8%)	14 (31.8%)	< 0.005
No	177 (95.2%)	30 (68.2%)	
Progression of cancer			
Yes	6 (3.2%)	7 (15.9%)	0.004
No	180 (96.8%)	37 (84.1%)	
Improvement in quality of life			
Yes	52 (28%)	35 (79.5%)	< 0.005
No	134 (72%)	9 (20.5%)	
Therapeutic obstinacy			
Yes	148 (79.6%)	22 (50%)	< 0.005
No	38 (20.4%)	22 (50%)	
Discontinuation of artificial nutrition for comfort			
Yes	101 (54.3%)	13 (29.5%)	0.004
No	85 (45.7%)	31 (70.5%)	

### Influence of the loss of patients’ decision-making capacity

When a physician or nurse showed personal convictions of the benefits of artificial nutrition at the end of life, the patient’s loss of decision-making capacity were not a motivator to withdraw or withhold artificial nutrition.

## Discussion

A significant majority of participants in this study indicated that they had encountered instances of withdrawing or withholding artificial nutrition at the end of life. Three-quarters of these participants opposed artificial nutrition and viewed it as an aggressive intervention that falls outside the scope of palliative care.

However, there was a notable variability in beliefs and knowledge regarding the prescription of artificial nutrition at the end of life. Participants commonly expressed perspectives on enhancing nutritional status, reducing broncho-aspiration events, preventing pressure sores, and improving the quality of life. This underscores the frequent occurrence and ongoing controversy surrounding the decision to withdraw or withhold artificial nutrition at the end of life in various settings, as previously noted in studies. Faber et al. found that a decision concerning withdrawal or waiver of artificial nutrition was present in 84% of deaths in an acute care setting [13], and Clarke et al. found such a decision in 65% of deaths (REF) [37]. These findings align with prior research despite the limited evidence available in the literature [2–6, 18, 38, 39]. A Cochrane meta-analysis by Good et al. found no impact on the quality and length of life [14]. However, it is essential to acknowledge variations among healthcare professionals with personal opinions either in favor of or against the use of artificial nutrition.

The existing literature on the beliefs and perceptions of healthcare professionals regarding artificial nutrition at the end of life is constrained [10, 11, 15–18, 40, 41]. Studies by Heuberger et al., Vitale et al., and Buiting et al. have highlighted that physicians may hold beliefs regarding the effectiveness of artificial nutrition in end-of-life such as a reduction in broncho-aspiration events or improvement in nutritional status that diverge from established evidence [10, 11, 40]. These findings prompt inquiries into the origins of these beliefs, which in our study persisted across regions, professions, prior exposure to artificial nutrition at the end of life, and years of professional experience. However, it is noteworthy that experience in palliative care significantly diminished these beliefs, except in Italian-speaking regions. Our findings suggest that the observed differences in the Italian-speaking region may be influenced by cultural and ethical perspectives on nutrition and care, including a stronger association of food with nurturing and support at the end of life. Additionally, variations in communication and decision-making dynamics, and differing patient and family expectations about artificial nutrition may play a significant role in shaping healthcare professionals’ attitudes and decisions in this region. Further research should enable us to identify additional factors that explain the results observed in Italian-speaking Switzerland. Additionally, physicians and nurses who were personally convinced of the benefits of artificial nutrition for themselves were more confident about its benefits for patients, even in patients lacking decision-making capacity.

Our study revealed that a significant majority of participants regarded artificial nutrition as a therapeutic measure. For most of them, the cessation of such nutrition was not viewed as neglect, assisted suicide, or a form of euthanasia. Nonetheless, it is important to acknowledge that 3.5% of participants perceived the withdrawal of artificial nutrition at the end of life as a form of assisted suicide and 3% as a form of euthanasia. Previous studies have indicated that irrespective of cultural factors, a lack of clinical knowledge can contribute to feelings of guilt and a fear of abandonment among healthcare professionals, potentially exacerbating concerns about the well-being of their patients [15–17, 41].

In the realm of decision-making studies, there has been a prevalence of cross-sectional approaches utilizing questionnaires, often neglecting considerations specific to decisions regarding artificial nutrition at the end of life [19–23, 42]. Contrary to the broader European context reported by Van der Heide et al. in 2001, wherein only 32% of participants exhibited similar sentiments, our study indicated slightly different outcomes in the Swiss regions [19]. Notably, in the Swiss context, Hurst et al. demonstrated a scarce involvement of patients in medical end-of-life decisions across all Swiss regions, with percentages of 16% in the French region, 31.2% in the German region, and 35.6% in the Italian region [43]. Despite these modest figures, existing data underscore the significance of considering the patient’s perspective in decision-making processes, as highlighted by many international studies [17, 20].

Over the last two decades, there has been a transformative shift in healthcare professional education from a paternalistic approach toward embracing autonomy and partnership with patients. In alignment with this evolving paradigm, our results concur with the emphasis on involving patients in decision-making and promoting patient autonomy. While our study underscores the importance of interprofessional decision-making with various healthcare professionals involved, it also prompts contemplation on how to incorporate the patient’s viewpoint into this process. This is particularly pertinent when the withdrawal of artificial nutrition is perceived as a complex issue, and healthcare professionals may even perceive a sense of neglect from the patient. In such situations, it is essential to consider how other ethical values, such as beneficence and non-maleficence may at times compete with the value of autonomy. For instance, our results indicate that beneficence and non-maleficence were more frequently reported as guiding principles in decision -making. These values can take precedence in cases where providing artificial nutrition is deemed more harmful than beneficial, even if patients or their caregivers are requesting it [5, 6, 36]. This raises the critical question of how to balance these ethical principles while maintaining a transparent and patient-centered approach in care.

The decision-making role of physicians and nurses varies based on the perceived significance of each in the decision-making process [17, 24]. For instance, Byron [23] demonstrated that the influence of nurses was constrained in the decision-making process, even though they played a substantial indirect role in initiating it. While nurses perceived their role as valuable in the decision, they also deemed that their responsibilities were not always clearly defined or appreciated.

The robustness of our study lies in its interprofessional and multicultural approach to the intricate subject of artificial nutrition at the end of life. The study’s strength is underscored by including a diverse panel of nurses and physicians from various settings and regions in Switzerland, facilitating a comprehensive spectrum of responses. However, there are several limitations to consider. Potential selection biases can be related to the voluntary participation to the survey, the risk of recruiting participants with a specific interest in the subject, and the majority of participants having less than 10 years of experience in clinical practice. Regarding potential response biases, the use of an anonymous and voluntary format mitigated this risk; however, we cannot exclude the possibility of a bias toward socially acceptable responses and, thus, the potential presence of a Hawthorne effect [44]. The response rate of 22% represents a limit in our study; however, we posit that certain factors may alleviate potential biases. The respondents can be deemed a representative sample of the population engaged in decisions regarding end-of-life artificial nutrition. Given the focus on general trends and beliefs, coupled with a meticulously designed questionnaire featuring clear and pertinent questions, the survey is positioned to yield valuable insight.

Despite these limitations, the strength of elucidating significant differences in beliefs and decision-making factors suggests the possibility of replicating this study on a larger scale to validate and reinforce the results. Investigating artificial nutrition and its effects on pressure sores, broncho-aspiration, and nutritional status is crucial for providing evidence and dispelling false beliefs. Furthermore, bolstering training in palliative care and implementing specific training programs at both pre- and post-graduate levels addressing this issue are expected to enhance professionals’ willingness to foster patient information and involvement in decisions.

## Conclusions

The decision-making process regarding artificial nutrition at the end of life is common among physicians and nurses and is mainly influenced by patient wishes and professional beliefs, often more than by solid evidence. Our study highlights the impact of palliative care experience on the alignment of decision-making with clinical recommendations.

## Supplementary Information

Below is the link to the electronic supplementary material. Questionnaire in French, in German, in Italian, in English.

ESM 1 (DOCX 32.9 KB)

ESM 2 (DOCX 32.5 KB)

ESM 3 (DOCX 36.7 KB)

ESM 4 (DOCX 25.0 KB)

## Data Availability

Data is available upon request.
