# Supplementary material for: The use of artificial nutrition at the end-of-life: a cross-sectional survey exploring the beliefs and decision-making among physicians and nurses
Source: Support Care Cancer. 2025 Mar 17;33(4):287. doi: 10.1007/s00520-025-09310-2 (PMC11914226; doi:10.1007/s00520-025-09310-2)
Supplement: Supplementary file 1 — (DOCX 32.9 KB) [file 520_2025_9310_MOESM1_ESM.docx]

**La nutrition artificielle en fin de vie**

**Questionnaire destiné au personnel médico-soignant**

Vous êtes invité-e à participer à une enquête multicentrique (Hôpitaux Universitaire de Genève, Inselspital de Bern, Hôpital cantonal du Tessin) menée par le service de médecine palliative des Hôpitaux Universitaires de Genève (HUG) dans le contexte d’un projet de thèse sur le thème de la nutrition artificielle en fin de vie.

Cette enquête a été soumise aux organes consultatifs des Hôpitaux Universitaire de Genève, de l’Inselspital et de l’Hôpital Cantonal du Tessin quant aux questions d’aspect éthique sur des projets de recherche conduits auprès des collaborateurs.

Le but de cette étude est d’évaluer les représentations en lien avec la nutrition artificielle en fin de vie et les questions relatives aux choix décisionnels dans les trois régions linguistiques principales de la Suisse.

Votre participation consiste à remplir un questionnaire qui ne devrait pas vous prendre plus que 15 minutes.

Votre participation est volontaire et anonyme puisqu’il n’y a aucun moyen de vous identifier, ni par les informations données, ni de façon informatique, l’adresse de votre ordinateur (adresse IP) n’étant pas enregistrée. De plus ces données sont stockées sur un serveur sécurisé des Hôpitaux Universitaires de Genève. Les résultats analysés peuvent faire l’objet de publications scientifiques. Toutes les personnes impliquées dans l’étude sont tenues au secret professionnel.

Nous espérons avoir retenu toute votre attention et vous remercions d’avance pour votre collaboration, en espérant grâce à votre aide pouvoir améliorer nos pratiques.

**Demande d’accord de consentement**

1. Avant de débuter les réponses au questionnaire, merci de valider votre consentement*

*Obligatoire

**Mise en situation**

Afin de pouvoir répondre aux questions ci-dessous, nous vous proposons une mise en situation avec le cas clinique de Madame C.

- **Motif d’hospitalisation :** progression de sa maladie oncologique métastatique et pneumonie d’aspiration sur fausse route.
- **Bilan fonctionnel**: nouvellement dépendante pour tous les AVQ (activité de la vie quotidienne) et AVQI (activité instrumentale de la vie quotidienne)
- **Motricité :** Elle est alitée la majorité du temps ou au fauteuil
- **Statut nutritionnel**: perte pondérale de > 15% en 1 mois, réduction de la prise alimentaire avec des fausses routes sur trouble de la déglutition.
- **Statut cognitif**: sans particularité.
- **Symptômes relevant :** Asthénie invalidante, somnolence, dyspnée stade III selon NYHA (New York Heart Association (classification de la dyspnée selon l’association de cardiologie de New York)), algies diffuses, fausses routes itératives, perte d’appétit
- **Echelle de performance PPSv2 (échelle de performance pour patients en soins palliatifs)** : 40-50% avec une espérance de vie théorique de 1 mois
- **Situation sociale**: veuve, vit seule, 2 enfants
- **Religion** : athée
- **Directives anticipées** : non présente
- **Attitude générale** : NTBR (Not to be resuscitated (ne pas réanimer))

**Définitions**

**Fin de vie** : Dans le cas présent nous définirons ce concept par une espérance de vie de l’ordre du mois avec une perte d’indépendance chez un patient avec une maladie oncologique sans trouble cognitif.

**Nutrition artificielle** : nutrition entérale par SNG ou PEG ou nutrition artificielle parentéral par accès veineux central ou périphérique

**Principes d’éthique :**

- **Autonomie**: respect pour chacun de disposer soi-même de sa santé et de choisir les options qui correspondent à ses souhaits et ses valeurs
- **Bienfaisance** : l’action de faire du bien ou le souci de vouloir le bien
- **Non-malfaisance** : l’action de ne pas nuire, ne pas causer d’effets indésirables
- **Justice** : l’obligation de traiter chacun de manière égale

**Négligence** : situation ou la décision de soin a été prise sans respecter les recommandations de bonnes pratiques et ayant causé un dommage à un individu.

**Suicide assisté** : pratique de fournir au patient la substance mortelle qu’il ingérera alors lui-même sans intervention extérieure, pour mettre fin à ses jours

**Euthanasie** : Administration par un tiers d’une substance dans le but de diminuer l’espérance de vie du patient

**Acharnement thérapeutique** : C’est le fait de pratiquer ou d’entreprendre des actes ou des traitements alors qu’ils apparaissent inutiles, disproportionnés ou n’ayant d’autre effet que le seul maintient artificiel de la vie

**Proche**: Dans le contexte, il s’agit de toute personne sans lien de sang mais ayant un rôle d’aidant auprès du patient

**Questions relatives au collectif interrogé**

1. Genre : H/F ?
2. Âge :
3. Langue régionale ?
4. Année.s d’expérience dans le milieu de la santé :
5. Profession : médecin ou infirmier-e ?
6. Service/Département :
7. Religion : Chrétienne/Juive/Musulmane/Bouddhiste/Hindouisme/ Pas de religion/ autres / ne souhaite pas répondre
8. Expérience en soins palliatifs spécialisés : oui/non ?
9. Si oui  Nombres d’années
10. Avez-vous déjà été confronté à une décision d’arrêt/renoncement de nutrition artificielle en fin de vie ? Oui ? non ?

**Questions générales sur la nutrition artificielle**

1. De manière générale, la nutrition artificielle est-elle selon vous une thérapie ou un soin de base ? au choix : thérapie/ soins de base ?
2. Quelles sont selon vous les indications de la nutrition artificielle chez Madame C.?

- Amélioration du statut nutritionnel ? oui/non
- Diminution des broncho aspirations ? oui/non
- Prévention des escarres ? oui/non
- Prévention de la sensation de soif ? oui/non
- Prévention de la sensation de faim ? oui/non
- Amélioration de l’asthénie ? oui/non
- Amélioration de son autonomie ? oui/non
- Amélioration de ses douleurs ? oui/non
- Retarde la progression oncologique ? oui/non
- Prolongation de la vie ? oui/non

1. De manière générale, la nutrition artificielle en fin de vie peut-elle selon vous :

- Améliorer la qualité de vie du patient ? oui/non

1. Si oui par quel mécanisme (réponse ouverte)
2. Et dans le cas de Madame C ?
3. Si une nutrition artificielle devait être débutée chez Madame C. puis arrêté cela pourrait être assimilé par vous à :

- De la négligence ? oui/non
- Un suicide assisté ? oui/non ?
- Une euthanasie ? oui/non

1. Le maintien de la nutrition artificielle en fin de vie peut-il être assimilé à :

- De l’acharnement thérapeutique ? oui/non ?

1. L’arrêt de la nutrition artificielle est-il nécessaire au confort du patient en fin de vie ? oui/non
2. La nutrition artificielle en fin de vie peut-il faire partie d’une approche palliative ?oui/non

**Questions sur le processus décisionnel**

1. L’instauration/l’arrêt/le renoncement d’une nutrition artificielle chez Madame C. sont-ils des sujets discutés de manière interprofessionnelle dans votre pratique ? oui/non
2. Avec quel professionnel partagez-vous la discussion ?

- Collègues médecins ? oui/non
- Collègues infirmier-e-s ? oui/non
- Collègues aide-soignant-e-s ? oui/non
- Autres professionnels ? oui/non

1. L’instauration/ l’arrêt/ le renoncement d’une nutrition artificielle dans le cas de Madame C. sont-ils des sujets à discuter :

- Seulement avec Madame C. ?oui/non
- Seulement avec ses proches ? oui/non
- Avec les deux ? oui/non

1. Si Madame C. est incapable de discernement ou ne peut communiquer, avec qui discutez-vous ?

- Proches
- Equipe interprofessionnelle
- Médecin traitant
- Aucun de ces choix

1. Quelle est selon vous l’importance de l’avis du médecin en charge dans la décision d’arrêt/renoncement de la nutrition artificielle en fin de vie ?  Peu/modérée/décisif ?
2. Quelle est selon vous l’importance de l’avis de l’infirmier-e dans la décision d’arrêt/ le renoncement de la nutrition artificielle en fin de vie ?  Peu/modérée/décisif ?
3. Quelle est selon vous l’importance de l’avis du patient dans la décision d’arrêt/ le renoncement de la nutrition artificielle en fin de vie ?  Peu/modérée/décisif ?
4. Quelle est selon vous l’importance des proches dans la décision d’arrêt/ le renoncement de la nutrition artificielle en fin de vie ? Peu/modérée/décisif ?
5. Quels sont les critères décisionnels que vous utilisez lors du début/ l’arrêt/ le renoncement de la nutrition artificielle :

- 1- L’espérance de vie du patient ? oui/non
- 2- La qualité de vie du patient ? oui/non
- 3- Le statut nutritionnel du patient ? oui/non
- 4- L’âge du patient ? oui/non
- 5- Les comorbidités ? oui/non

1. En lien avec la question 29, grader l’importance de ces critères

- 1- L’espérance de vie du patient ? faible/modérée/fort
- 2- La qualité de vie du patient ? faible/modérée/fort
- 3- Le statut nutritionnel du patient ? faible/modérée/fort
- 4- L’âge du patient ? faible/modérée/fort
- 5- Les comorbidités ? faible/modérée/fort

1. Dans le cas de Madame C. quels critères vous pousseraient à débuter une nutrition artificielle ? (texte libre)
2. Dans le cas de Madame C. quels critères vous amèneraient à renoncer à une nutrition artificielle ? (texte libre)
3. Quel principe éthique influence votre décision d’arrêter la nutrition artificielle ?

- Le principe de Justice ? oui/non
- Le principe de bienfaisance ? oui/non ?
- Le principe de non-malfaisance ? oui/non
- Le principe d’autonomie ? oui/non

1. En lien avec la question 33, grader l’importance de ces critères

- Le principe de Justice ?
- Le principe de bienfaisance ?
- Le principe de non-malfaisance ?
- Le principe d’autonomie ?

1. Si vous étiez dans la situation de Madame C. seriez-vous pour ou contre l’usage de nutrition artificielle ? pour/contre
2. Si vous deviez perdre votre capacité de discernement seriez-vous toujours pour ou contre ?
3. Quels niveaux de certitudes avez-vous si vous deviez prendre la décision de débuter une nutrition artificielle chez Madame C. ?  Faible/modéré/fort
4. Quels niveaux de certitudes avez-vous si vous deviez prendre la décision de renoncer à une nutrition artificielle chez Madame C. ?  Faible/modéré/fort
5. Que feriez-vous si Madame C. n’est pas d’accord avec votre décision ?

- Acceptez-vous son avis ? Oui/non
- Ne pas prendre en compte son avis ? oui/non
- Organiser une réunion avec ses proches ? oui/non
- Organiser une réunion interprofessionnelle ? oui/non
- Demander l’avis du conseil d’éthique ? oui/non

1. Si un désaccord décisionnel intervient dans votre équipe que faites-vous ?

- Vous suivez la majorité ? oui/non
- Vous suivez l’avis du patient ?oui/non
- Vous suivez l’avis des proches ? oui/non
- Vous consultez le conseil d’éthique ? oui/non

1. Après avoir débuté une nutrition artificielle prévoyez-vous de réévaluer son usage ? oui/non
2. La nécessité de réévaluation de la nutrition artificielle était-elle discutée avec votre patient avant son instauration ? oui/non
3. Et avec ses proches ? oui/non
4. Avec qui effectuez-vous la réévaluation ? :

- Patient oui/non
- Proches oui/non
- Collègues médecins oui/non
- Collègues Infirmier-e-s oui/non
- Diététicien-ne oui/non

1. Quels critères utilisez-vous pour réévaluer la nutrition artificielle ? : (texte libre)
