# Supplementary material for: The use of artificial nutrition at the end-of-life: a cross-sectional survey exploring the beliefs and decision-making among physicians and nurses
Source: Support Care Cancer. 2025 Mar 17;33(4):287. doi: 10.1007/s00520-025-09310-2 (PMC11914226; doi:10.1007/s00520-025-09310-2)
Supplement: Supplementary file 2 — (DOCX 32.5 KB) [file 520_2025_9310_MOESM2_ESM.docx]

**Fragebogen für Ärzte und Pflegefachpersonen : Künstliche Ernährung am Lebensende**

Sie sind eingeladen, an einer multizentrischen Umfrage (Universitätsspital Genf, Inselspital Bern, Kantonsspital Tessin) teilzunehmen, welche von der Abteilung für Palliativmedizin des Universitätsspitals Genf (HUG) im Rahmen einer Doktorarbeit zum Thema künstliche Ernährung am Lebensende durchgeführt wird. Diese Umfrage erhielt eine Ausnahmebewilligung der lokalen Kommission (HUG) für akademische Ausbildung und Forschung in der Pflege. Das Ziel dieser Studie ist es, die Einstellungen im Zusammenhang mit künstlicher Ernährung am Lebensende zu beurteilen und zu erfassen, und zu erfahren mit welchen Fragen sich das Pflegepersonal in der Entscheidungsfindung in den drei Hauptsprachregionen der Schweiz auseinandersetzt.

Für das Ausfüllen des Fragebogens benötigen Sie voraussichtlich 15 Minuten. Es steht Ihnen frei, an der Umfrage teilzunehmen oder nicht. Die zu Forschungszwecken erhobenen Daten sind anonym. Die analysierten Ergebnisse können in wissenschaftlichen Publikationen veröffentlicht werden. Die Daten werden vertraulich behandelt und Ihr Name oder andere Informationen, die Sie identifizieren könnten, werden nicht publiziert. Alle an der Studie beteiligten Personen sind dem Berufsgeheimnis unterstellt.

Wir hoffen, dass wir Ihr Interesse geweckt haben. Wir danken Ihnen im Voraus für Ihre Teilnahme und hoffen, dass wir mit Ihrer Hilfe unsere Praxis verbessern können.

**Freiwillige Teilnahmebestätigung**

1. Bevor Sie mit der Beantwortung des Fragebogens beginnen, bestätigen Sie bitte Ihre Zustimmung

- Ich bestätige meine Einwilligung

**Ein Praxisbeispiel**

Um die unten stehenden Fragen beantworten zu können, schlagen wir Ihnen das Fallbeispiel von Frau C. vor:

- **Grund der Spitaleinweisung:** Fortschreiten einer metastasierenden onkologischen Erkrankung und Pneumonie durch Aspiration von Nahrungsmitteln.
- **Mobilität:** die Patientin braucht seit kurzem Hilfe für sämtliche Aktivitäten des täglichen Lebens (ATL) sowie für alle instrumentellen Aktivitäten des täglichen Lebens (IADL).
- **Beweglichkeit:** Sie bewegt sich unabhängig vom Bett auf den Stuhl, weitere körperliche Aktivitäten sind nicht möglich.
- **Ernährungsstatus:** Gewichtsverlust von > 15 % in 1 Monat, reduzierte Nahrungsaufnahme mit Schluckproblemen und häufiger Inhalation von Nahrungsmitteln.
- **Kognitiver Status:** ohne Besonderheiten
- **Haupt-Symptome:** stark beinträchtigende Asthenie, Schläfrigkeit, Dyspnoe Stadium III nach NYHA, diffuse Schmerzen, Schluckprobleme mit häufiger Inhalation von Nahrungsmitteln, Appetitlosigkeit.
- **PPSv2-Palliative Performance Scale:** 40-50% theoretische Lebenserwartung von 1 Monat
- **Soziale Situation:** verwitwet, lebt alleine, 2 Kinder
- **Religion:** konfessionslos
- **Patientenverfügung:** nicht vorhanden
- **Allgemeiner Code Status:** NTBR (Not to be resuscitated oder nicht reanimieren)

**Definitionen**

**Lebensende:** Im vorliegenden Fall definieren wir diesen Begriff durch eine Lebenserwartung in der Größenordnung von einem Monat mit gleichzeitigem Autonomie-Verlust bei einem Patienten mit einer onkologischen Erkrankung ohne kognitive Beeinträchtigung.

**Künstliche Ernährung:** Enterale Ernährung über Nasen- oder PEG-Sonde oder parenterale künstliche Ernährung über einen zentralen oder peripheren Venenzugang.

**Ethischen Grundlagen**:

- Autonomie/Selbstbestimmungsrecht: Respekt für jeden Menschen, selbst über seine Gesundheit zu bestimmen und die Optionen zu wählen, die seinen Wünschen und Werten entsprechen.
- Patientenwohl: ist die Handlung, Gutes zu tun, oder die Sorge, Gutes zu wollen.
- Schadensvermeidung: ist die Handlung, nicht zu schaden, keine unerwünschten Wirkungen zu verursachen.
- Soziale Gerechtigkeit: ist die Verpflichtung, jeden gleich zu behandeln

**Vernachlässigung :** Situation, in welcher eine Entscheidung getroffen wurde, ohne Beachtung der Praxisempfehlungen und welche der Person einen Schaden zugefügt hat.

**Suizidhilfe** : Die Praxis, einem Patienten eine tödliche Substanz zu verabreichen, die er dann selbst ohne äußere Einwirkung einnimmt, um sein Leben zu beenden

**Euthanasie:** Die Verabreichung einer Substanz durch eine Drittperson mit dem Ziel, das Leben zu beenden.

**Lebensverlängernde Massnahmen** : Die Durchführung oder Einleitung von Handlungen oder Behandlungen, die unnötig oder unverhältnismäßig erscheinen oder keine andere Wirkung haben als die künstliche Aufrechterhaltung des Lebens.

**Familie:** In diesem Zusammenhang ist jede Person gemeint, die mit dem Patienten blutsverwandt ist.

**Angehörige:** In diesem Zusammenhang sind dies alle Personen, die nicht blutsverwandt sind, aber eine helfende Rolle für den Patienten haben.

**Fragen zu ihrer Person**

1. Geschlecht: M/F
2. Alter
3. Muttersprache
4. Anzahl Jahre Berufserfahrung im Gesundheitsbereich
5. Beruf: Arzt/Ärztin oder Pflegefachperson
6. Abteilung/Klinik
7. Religion:

- Christentum
- Judentum
- Islam
- Buddhismus
- Hinduismus
- Keine Religion
- Sonstige
- Ich möchte darauf nicht antworten

1. Erfahrung in spezialisierter Palliativversorgung: ja/nein?
2. Wenn ja, Anzahl Jahre.
3. Wurden Sie jemals mit der Entscheidung konfrontiert, künstliche Ernährung am Lebensende zu beenden oder zu unterlassen? Ja? Nein?

**Allgemeine Fragen zur künstlichen Ernährung**

1. Ist künstliche Ernährung Ihrer Meinung nach generell eine Therapie oder gehört diese zur Grundpflege? zur Auswahl: Therapie/Grundpflege?
2. Was sind Ihrer Meinung nach die Indikationen für eine künstliche Ernährung bei Frau C.?

- Verbesserung des Ernährungsstatus? ja/nein
- Abnahme der Aspirationspneumonien? ja/nein
- Vorbeugung von Druckgeschwüren? ja/nein
- Vermeidung von Durstgefühl? ja/nein
- Vermeidung von Hungergefühl? ja/nein
- Verbesserung der Asthenie? ja/nein
- Verbesserung der Selbstständigkeit? ja/nein
- Verbesserung der Schmerzen? ja/nein
- Verzögerung des Fortschreiten der onkologischen Erkrankung? ja/nein
- Verlängerung des Lebens? ja/nein

1. Kann die künstliche Ernährung am Lebensende Ihrer Meinung nach generell:

- die Lebensqualität des Patienten verbessern? Ja/Nein

1. Falls ja durch welchen Mechanismus (freier Text).
2. Und im Fall von Frau C.? (freier Text)
3. Wenn bei Frau C. eine künstliche Ernährung begonnen und danach wieder eingestellt wird, könnte dies von Ihnen als Folgendes aufgefasst werden:

- Als Vernachlässigung? ja/nein
- Als assistierter Suizid? ja/nein?
- Als Euthanasie? ja/nein

1. Könnte die Aufrechterhaltung der künstlichen Ernährung am Lebensende von Ihnen gleichgesetzt werden mit:

- Lebensverlängernden Massnahmen? ja/nein?

1. Ist die Beendigung der künstlichen Ernährung für das Wohlbefinden des Patienten am Lebensende notwendig? ja/nein
2. Ist die künstliche Ernährung eine palliative Behandlung? ja/nein

**Fragen zum Entscheidungsfindungsprozess**

1. Ist die Einleitung/Beendigung/Verzicht auf künstliche Ernährung, wie z.B. bei Frau C. ein Thema, das in Ihrer Praxis interprofessionell diskutiert wird? ja/nein
2. Mit welchen Fachpersonen teilen Sie die Diskussion?

- Ärztliche Kollegen/Innen? ja/nein
- Kollegen der Krankenpflege? ja/nein
- Kollegen aus weiteren Pflegebereichen? ja/nein
- Andere Fachpersonen? ja/nein

1. Ist die Einleitung/Beendigung/Verzicht auf künstliche Ernährung im Fall von Frau C. ein Thema, welches mit folgenden Personen besprochen werden sollte:

- nur mit Frau C.? ja/nein
- nur mit den Angehörigen von Frau C.? ja/nein
- mit beiden? ja/nein

1. Wenn Frau C. urteilsunfähig ist oder nicht kommunizieren kann, mit wem besprechen Sie das Thema? Mehrfachauswahl:

- Angehörige?
- Interprofessionelles Team?
- Der Hausarzt?
- Keine der obigen Antworten trifft zu?

1. Wie wichtig ist Ihrer Meinung nach der Standpunkt des zuständigen Arztes bei der Entscheidung über die Beendigung/den Verzicht auf künstliche Ernährung am Lebensende?

- Wenig
- Mittelmässig
- Entscheidend

1. Wie wichtig ist Ihrer Meinung nach der Standpunkt der zuständigen Pflegefachperson bei der Entscheidung über die Beendigung/den Verzicht auf künstliche Ernährung am Lebensende?

- Wenig
- Mittelmässig
- Entscheidend

1. Wie wichtig ist Ihrer Meinung nach der Standpunkt des Patienten/in bei der Entscheidung über die Beendigung/den Verzicht auf künstliche Ernährung am Lebensende?

- Wenig
- Mittelmässig
- Entscheidend

1. Wie wichtig ist Ihrer Meinung nach der Standpunkt der Familie bei der Entscheidung über die Beendigung/den Verzicht auf künstliche Ernährung am Lebensende?

- Wenig
- Mittelmässig
- Entscheidend

1. Welche Entscheidungskriterien verwenden Sie beim Beginn/bei der Beendigung/beim Verzicht auf künstliche Ernährung:

- Die Lebenserwartung des Patienten? ja/nein
- Die Lebensqualität des Patienten? ja/nein
- Der Ernährungsstatus des Patienten/in? ja/nein
- Das Alter des Patienten/in? ja/nein
- Die Komorbiditäten? ja/nein

1. Bitte gewichten Sie dieses Kriterium in Bezug auf die Wichtigkeit bei der Entscheidungsfindung: schwach/mittelmässig/stark.

- Die Lebenserwartung des Patienten? schwach/mittelmässig/stark.
- Die Lebensqualität des Patienten? schwach/mittelmässig/stark.
- Der Ernährungsstatus des Patienten/in? schwach/mittelmässig/stark.
- Das Alter des Patienten/in? schwach/mittelmässig/stark.
- Die Komorbiditäten? schwach/mittelmässig/stark.

1. Welche Kriterien würden Sie im Fall von Frau C. dazu veranlassen, mit einer künstlichen Ernährung zu beginnen? (freier Text)
2. Welche Kriterien würden Sie im Fall von Frau C. dazu veranlassen, auf eine künstliche Ernährung zu verzichten? (freier Text)
3. Welches ethische Prinzip beeinflusst Ihre Entscheidung, die künstliche Ernährung zu beenden?

- Das Prinzip der sozialen Gerechtigkeit? ja/nein
- Das Prinzip des Patientenwohls? ja/nein
- Das Prinzip der Schadensvermeidung? ja/nein
- Das Prinzip der Autonomie/Selbstbestimmung? ja/nein

1. Bitte gewichten Sie dieses Kriterium in Bezug auf die Wichtigkeit bei der Entscheidungsfindung

- Das Prinzip der sozialen Gerechtigkeit? schwach/mittelmässig/stark.
- Das Prinzip des Patientenwohls? schwach/mittelmässig/stark.
- Das Prinzip der Schadensvermeidung? schwach/mittelmässig/stark.
- Das Prinzip der Autonomie/Selbstbestimmung? schwach/mittelmässig/stark.

1. Wenn Sie in der Situation von Frau C. wären, wären Sie für oder gegen die Verwendung von künstlicher Ernährung? dafür/dagegen
2. Wenn Frau C. ihre Urteilsfähigkeit verlieren würden, wären Sie dann immer noch dafür oder gegen eine künstliche Ernährung? dafür/dagegen
3. Wie sicher wären Sie, wenn Sie die Entscheidung treffen müssten, bei Frau C. mit einer künstlichen Ernährung zu beginnen? gering/mittelmässig/stark
4. Wie sicher wären Sie, wenn Sie die Entscheidung treffen müssten, bei Frau C. auf eine künstliche Ernährung zu verzichten? gering/mittel mässog/stark
5. Was würden Sie tun, wenn Frau C. mit Ihrer Entscheidung nicht einverstanden ist?

- Würden Sie den Standpunkt von Frau C akzeptieren? Ja/Nein
- Würden Sie den Standpunkt von Frau C nicht berücksichtigen? Ja/Nein
- Organisieren Sie eine Besprechung mit Frau C und ihrer Familie? Ja/Nein
- Organisieren Sie eine interprofessionelle Teamsitzung? Ja/Nein
- Holen Sie die Meinung des Ethikrates ein?

1. Wenn in Ihrem Team Uneinigkeit bezüglich einer Entscheidungsfindung besteht, was unternehmen Sie?

- Folgen Sie der Mehrheit?
- Folgen Sie des Standpunktes des Patienten?
- Folgen Sie des Standpunktes der Familie?
- Konsultieren Sie den Ethikrat?

1. Planen Sie nach Beginn einer künstlichen Ernährung eine Neubewertung deren Weiterführung? ja/nein
2. Wurde die Notwendigkeit einer Neubewertung der künstlichen Ernährung mit Ihrem/Ihrer Patienten/in vor Beginn der künstlichen Ernährung besprochen? ja/nein und mit den Angehörigen? ja/nein
3. Und mit seinen Liebsten? Ja/Nein
4. Mit wem führen Sie die Neubewertung durch?

- Patient/in ja/nein
- Angehörige ja/nein
- Ärztekollegen/innen ja/nein
- Kollegen aus dem Pflegefachbereich ja/nein
- Ernährungsberater/in ja/nein

1. Welche Kriterien verwenden Sie, um die künstliche Ernährung neu zu bewerten? (freier Text)
