# Supplementary material for: The use of artificial nutrition at the end-of-life: a cross-sectional survey exploring the beliefs and decision-making among physicians and nurses
Source: Support Care Cancer. 2025 Mar 17;33(4):287. doi: 10.1007/s00520-025-09310-2 (PMC11914226; doi:10.1007/s00520-025-09310-2)
Supplement: Supplementary file 3 — (DOCX 36.7 KB) [file 520_2025_9310_MOESM3_ESM.docx]

**Questionario Nutrizione artificiale di fine vita destinata al personale medico e infermieristico**

**Nutrizione artificiale di fine vita destinata al personale medico e infermieristico**

**Siete invitati a partecipare ad un sondaggio multicentrico (Ospedali Universitari di Ginevra, Inselspital di Berna, Ospedale Cantonale del Ticino) condotto dal Dipartimento di Medicina Palliativa degli Ospedali Universitari di Ginevra (HUG) nell'ambito di un progetto di tesi sul tema della nutrizione artificiale alla fine della vita.**

**Questa indagine è stata sottoposta agli organi consultivi degli Ospedali Universitari di Ginevra, dell'Insespital di Berna e dell'Ospedale Cantonale del Ticino per quanto riguarda gli aspetti etici dei progetti di ricerca condotti con il loro personale. Lo scopo di questo studio è quello di valutare le rappresentazioni relative alla nutrizione artificiale alla fine della vita e le questioni relative al processo decisionale nelle tre principali regioni linguistiche della Svizzera.**

**La sua partecipazione consiste nel riempire un questionario che dovrebbe richiedere solo 15 minuti. La sua partecipazione è volontaria e anonima poiché non c'è modo di identificarti, né dalle informazioni fornite né dal computer, dato che l'indirizzo del tuo computer (indirizzo IP) non viene registrato. Inoltre, questi dati sono conservati su un server sicuro degli Ospedali Universitari di Ginevra. I risultati analizzati possono essere utilizzati per pubblicazioni scientifiche. Tutte le persone coinvolte nello studio sono tenute al segreto professionale.**

**Speriamo di aver tratenuto la sua attenzione e la ringraziamo in anticipo per la sua collaborazione, sperando di migliorare le nostre pratiche con il suo aiuto.**

**Domanda di consenso**

1. Prima di iniziare a rispondere al questionario, la preghiamo di convalidare il suo consenso

Io convalido

*Obligatoio

Per poter rispondere alle domande che seguono, proponiamo un caso di studio della signora C.

**Motivo del ricovero:**progressione della sua malattia oncologica metastatica e polmonite da aspirazione dovuta a una falsa via.
**Valutazione funzionale:** nuova dipendenza per tutte le ADL (attività della vita quotidiana) e IADL (attività strumentale della vita quotidiana)
**Capacità motorie:**È costretta a letto per la maggior parte del tempo o su una sedia a rotelle
**Stato nutrizionale:** perdita di peso di > 15% in 1 mese, riduzione dell'assunzione di cibo sul disturbo della deglutizione
**Stato cognitivo:** nessuna caratteristica speciale
**Sintomi rilevanti:** Astenia invalidante, sonnolenza, dispnea stadio NYHA III, dolore diffuso, malaria ricorrente, perdita di appetito
**Scala delle prestazioni** **PPSv2 (Scala di rendimento per i pazienti in cure palliative)**: 40-50% di 1 mese
**Situazione sociale:** vedova, vive da sola, 2 figli
**Religione**: Ateo
**Direttiva anticipata:** non presente
**Atteggiamento generale:**NTBR (Not to be resuscitated o non essere rianimato)

**Definizione di fine vita:** in questo caso definiremo questo concetto con un'aspettativa di vita dell'ordine di un mese con una perdita di indipendenza in un paziente con una malattia oncologica senza deterioramento cognitivo.

**Definizione di nutrizione artificiale:** nutrizione enterale tramite SNG o PEG o nutrizione artificiale parenterale tramite accesso venoso centrale o periferico

**Definizione di principi etici :**

- **Autonomia:** rispetto del diritto di ogni persona a gestire la propria salute e a scegliere le opzioni che corrispondono ai suoi desideri e valori
- **Benevolenza:** è l'azione di fare del bene o la preoccupazione di fare del bene
- **Non-maleficenza:** è l'azione di non nuocere, non causare effetti negativi
- **Giustizia:** è l'obbligo di trattare tutti allo stesso modo

**Negligenza**: una situazione in cui una decisione di cura è stata presa senza seguire le raccomandazioni di buona pratica e ha causato un danno a un individuo.
**Suicidio assistito**: La pratica di fornire al paziente la sostanza letale, che poi ingerisce da solo senza intervento esterno, per porre fine alla sua vita.
**Eutanasia:** somministrazione da parte di terzi di una sostanza con lo scopo di abbreviare l'aspettativa di vita del paziente.
**Accanimento terapeutico:**è la pratica o l'esecuzione di atti o trattamenti quando appaiono inutili, sproporzionati o che non hanno altro effetto che il mantenimento artificiale della vita.
**Famiglia**: nel contesto, si tratta di qualsiasi persona con una relazione di sangue con il paziente.
**Parente:** Nel contesto, si tratta di qualsiasi persona senza una relazione di sangue ma con un ruolo di cura con il paziente.

**Domande relative al collettivo intervistato**

1. Sesso Uomo/Donna
2. Età
3. Lingua madre
4. Anno-i di esperienza nel settore sanitario
5. Professione
6. Servizio/Dipartimento
7. Religione

- Cristiana
- Ebraica
- Musulmana
- Buddista
- Induista
- Nessuna religione
- Non desidero rispondere

1. Esperienza in cure palliative specialistiche

- Sì
- No

1. Se sì, da quanti anni ?
2. Vi siete mai trovati di fronte alla decisione di fermare/togliere la nutrizione artificiale alla fine della vita?

- Sì
- No

Domande generali sulla nutrizione artificiale

1. In generale, considera la nutrizione artificiale una terapia o una cura di base? Terapie/ Cura di base ?
2. Quali sono secondo lei le indicazioni per la nutrizione artificiale nella signora C.?

- Miglioramento dello stato nutrizionale? Sì/No
- Diminuzione della broncoaspirazione? Sì/No
- Prevenzione delle piaghe da decubito? Sì/No
- Prevenzione della sete? Sì/No
- Prevenzione della fame? Sì/No
- Miglioramento dell'astenia? Sì/No
- Maggiore autonomia? Sì/No
- Miglioramento del dolore? Sì/No
- Ritarda la progressione oncologica? Sì/No
- Estensione della vita? Sì/No

1. In generale, pensa che la nutrizione artificiale alla fine della vita possa :

- Migliorare la qualità della vita del paziente? Sì/No
- E nel caso della signora C? Sì/No

1. Se sì, con quale meccanismo? Altrimenti scrivi no.
2. E nel caso dalla signora C?
3. Se la nutrizione artificiale dovesse essere iniziata per la signora C. e poi interrotta, questo potrebbe essere considerato da voi come :

- Negligenza? Sì/No
- Suicidio assistito? Sì/No
- Eutanasia? Sì/No

1. Il mantenimento della nutrizione artificiale alla fine della vita può essere equiparato a :

- Accanimento terapeutico? Sì/No

1. L'interruzione della nutrizione artificiale è necessaria per il comfort del paziente alla fine della vita? Sì/No
2. La nutrizione artificiale è un trattamento di gestione palliativa? Sì/No
3. L'inizio/l'arresto/il ritiro della nutrizione artificiale per la signora C. è un argomento discusso a livello interprofessionale nella sua pratica?Sì/No
4. Con quale professionista condivide la discussione?

- Colleghi medici? Sì/No
- Colleghi infermieri? Sì/No
- Colleghi caregiver? Sì/No
- Altri professionisti? Sì/No

1. L'inizio/arresto/interruzione della nutrizione artificiale nel caso della signora C. è una questione da discutere?

- solo con la signora C.? Sì/No
- solo con i parenti? Sì/No
- con entrambi? Sì/No

1. Se lei fosse nella situazione della signora C. e perdesse la sua capacità di discernimento, sarebbe favorevole o contrario alla nutrizione artificiale?

- Parenti?
- Squadra interprofessionale?
- Medico di famiglia
- Nessuna di queste scelte?

1. Quanto pensa sia importante l'opinione del medico curante nella decisione di fermare/ di togliere la nutrizione artificiale alla fine della vita? Poco/Moderato/Decisivo
2. Quanto pensa sia importante l'opinione dell'infermiere nella decisione di interrompere/ di togliere la nutrizione artificiale alla fine della vita? Poco/Moderato/Decisivo
3. Quanto pensa sia importante l'opinione del paziente nella decisione di interrompere/ di togliere la nutrizione artificiale alla fine della vita? Poco/Moderato/Decisivo
4. Quanto pensa che la famiglia sia importante nella decisione di fermare/togliere la nutrizione artificiale alla fine della vita? Poco/Moderato/Decisivo
5. Quali sono i criteri di decisione che usa quando inizia/interrompe/interrompe la nutrizione artificiale?

- Aspettativa di vita del paziente? Sì/No
- Qualità della vita del paziente? Sì/No
- Stato nutrizionale del paziente? Sì/No
- Età del paziente? Sì/No
- Comorbilità? Sì/No

1. In relazione alla domanda 29, classificate l'importanza di questi criteri

- Aspettativa di vita del paziente? Basso/Moderato/Forte
- Qualità della vita del paziente? Basso/Moderato/Forte
- Stato nutrizionale del paziente? Basso/Moderato/Forte
- Età del paziente? Basso/Moderato/Forte
- Comorbilità? Basso/Moderato/Forte

1. Nel caso della signora C., quali criteri la porterebbero a iniziare la nutrizione artificiale?
2. Nel caso della signora C., quali criteri la porterebbero ad abbandonare la nutrizione artificiale?
3. Quali principi etici influenzano la sua decisione di interrompere la nutrizione artificiale?

- 1- Il principio di giustizia? Sì/No
- 2- Il principio di beneficenza? Sì/No
- 3- Il principio di non-maleficenza? Sì/No
- 4- Il principio di autonomia? Sì/No

1. In relazione alla domanda 33, classificate l'importanza di questi criteri

- 1- Il principio di giustizia? Basso/Moderato/Forte
- 2- Il principio di beneficenza? Basso/Moderato/Forte
- 3- Il principio di non-maleficenza? Basso/Moderato/Forte
- 4- Il principio di autonomia? Basso/Moderato/Forte

1. Se lei fosse nella situazione della signora C, sarebbe favorevole o contrario all'uso della nutrizione artificiale? A favore/Contro
2. Se lei dovesse perdere la sua capacità di discernimento sarebbe ancora a favore o contro? A favore/Contro
3. Quali livelli di certezza ha se dovesse prendere la decisione di iniziare la nutrizione artificiale per la signora C.? Basso/Moderato/Forte
4. Quali livelli di certezza ha se dovesse prendere la decisione di ritirare la nutrizione artificiale alla signora C.? Basso/Moderato/Forte
5. Cosa farebbe se la signora C. non fosse d'accordo con la sua decisione?

- Accetta la sua opinione? Sì/No
- Non prendere in considerazione la sua opinione? Sì/No
- Organizzare un incontro con la sua famiglia? Sì/No
- Organizzare un incontro interprofessionale? Sì/No
- Chiedere consiglio al consiglio etico? Sì/No

1. Se c'è un disaccordo decisionale nella tua squadra, cosa fai?

- Segui la maggioranza? Sì/No
- Segui i consigli del paziente? Sì/No
- Segui i consigli della famiglia? Sì/No
- Consultate il consiglio etico? Sì/No

1. Dopo aver iniziato la nutrizione artificiale prevede di rivalutarne l'uso? Sì/No
2. La necessità di rivalutare la nutrizione artificiale è stata discussa con il paziente prima del suo inizio? Sì/No
3. E con la famiglia del paziente Sì/No
4. Con chi si effettua la rivalutazione?

- Paziente Sì/No
- Con la famiglia Sì/No
- Colleghi medici Sì/No
- Colleghi Infermiere Sì/No
- Dietista Sì/No

1. Quali criteri usate per rivalutare la nutrizione artificiale?
