# Supplementary material for: The use of artificial nutrition at the end-of-life: a cross-sectional survey exploring the beliefs and decision-making among physicians and nurses
Source: Support Care Cancer. 2025 Mar 17;33(4):287. doi: 10.1007/s00520-025-09310-2 (PMC11914226; doi:10.1007/s00520-025-09310-2)
Supplement: Supplementary file 4 — (DOCX 25.0 KB) [file 520_2025_9310_MOESM4_ESM.docx]

**Artificial nutrition at the end of life**

**Questionnaire for medical and nursing staff**

You are invited to take part in a multi-centre survey (University Hospitals of Geneva, Inselspital Bern, Cantonal Hospital of Ticino) conducted by the Palliative Medicine Department of the University Hospitals of Geneva (HUG) as part of a thesis project on the subject of artificial nutrition at the end of life.

The survey was submitted to the advisory bodies of the University Hospitals of Geneva, the Inselspital and the Cantonal Hospital of Ticino for their views on the metical aspects of research projects involving their staff.

The aim of this study is to evaluate the representations associated with artificial nutrition at the end of life and the issues relating to decision-making choices in Switzerland's three main linguistic regions.

Your participation consists of completing a questionnaire which should take no more than 15 minutes.

Your participation is voluntary and anonymous, as there is no way of identifying you, either through the information you provide or by computer, as your computer's address (IP address) is not recorded. In addition, this data is stored on a secure server at Geneva University Hospitals. The results analysed may be the subject of scientific publications. All those involved in the study are bound by professional secrecy.

We hope we've caught your attention and thank you in advance for your cooperation. We hope we can improve our practices with your help.

**Request for consent**

1. Before starting to answer the questionnaire, please validate your consent*.

*Mandatory

**Case scenario**

To help you answer the questions below, we'd like to give you an example of a clinical situation involving Mrs C.

- **Reason for hospitalisation:** progression of her metastatic oncological disease and aspiration pneumonia due to a false route.
- **Functional assessment:** newly dependent for all ADLs (activities of daily living) and IADLs (instrumental activities of daily living).
- **Motor skills:** she is bedridden most of the time or in a wheelchair.
- **Nutritional status:** weight loss of > 15% in 1 month, reduced food intake with false routes due to swallowing problems.
- **Cognitive status:** unremarkable.
- **Relevant symptoms:** Disabling asthenia, somnolence, dyspnoea NYHA stage III (New York Heart Association classification of dyspnoea), diffuse pain, iterative false swallowing, loss of appetite.
- **PPSv2 performance scale (performance scale for palliative care patients):** 40-50% with a theoretical life expectancy of 1 month
- **Social status:** widow, lives alone, 2 children
- **Religion:** atheist
- **Advance directives:** not present
- **General attitude:** NTBR (Not to be resuscitated)

**Definitions**

**End of life:** In this case, we will define this concept as a life expectancy of around one month with a loss of independence in a patient with an oncological disease without cognitive impairment.

**Artificial nutrition:** enteral nutrition via SNG or PEG or parenteral artificial nutrition via central or peripheral venous access.

**Ethical principles :**

- **Autonomy:** respect for each person's right to manage their own health and to choose the options that correspond to their wishes and values.
- **Beneficence:** the action of doing good or the desire to do good
- **Non-maleficence:** the action of not causing harm or undesirable effects
- **Justice:** the obligation to treat everyone equally

**Negligence:** a situation where a care decision has been taken without respecting the recommendations of good practice and has caused harm to an individual.

**Assisted suicide:** the practice of providing patients with a lethal substance, which they then ingest themselves without outside intervention, to end their lives.

**Euthanasia:** Administration by a third party of a substance with the aim of shortening the patient's life expectancy.

**Therapeutic overkill:** The practice or undertaking of acts or treatments when they appear to be useless, disproportionate or having no other effect than the artificial maintenance of life.

**Relative:** In this context, any person who is not related by blood but who has a caring role with the patient.

**Questions relating to the group surveyed**

2. Gender: M/F?

3. Age ?

4. Regional language ?

5. Years of experience in the health sector :

6. Profession: doctor or nurse?

7. Department :

8. Religion: Christian/Jewish/Muslim/Buddhist/Hindu/ No religion/ other/ do not wish to answer

9. Experience in specialist palliative care: yes/no?

10. If yes Number of years

11. Have you ever been confronted with a decision to stop/withdraw artificial nutrition at the end of life? Yes ? No ?

**General questions on artificial nutrition**

12. Generally speaking, do you consider artificial nutrition to be therapy or basic care?

13. In your opinion, what are the indications for artificial nutrition for Mrs C.?

- Improvement in nutritional status? yes/no
- Reduction in bronchoaspiration? yes/no
- Prevention of bedsores? yes/no
- Prevention of thirst? yes/no
- Prevention of hunger? yes/no
- Improve asthenia? yes/no
- Improved autonomy? yes/no
- Improves pain? yes/no
- Delays oncological progression? yes/no
- Prolong life? yes/no

14. Generally speaking, in your opinion, can artificial nutrition at the end of life :

- Improve the patient's quality of life? yes/no

15. If yes, by what mechanism (open answer)

16. And in the case of Mrs C?

17. If artificial nutrition were to be started for Mrs C. and then stopped, would you consider this to be :

- Neglect? yes/no

- Assisted suicide? yes/no

- Euthanasia? yes/no

18. Can maintaining artificial nutrition at the end of life be considered as :

- Therapeutic obstinacy? yes/no

19. Is stopping artificial nutrition necessary for the comfort of the patient at the end of life? yes/no

20. Can artificial nutrition at the end of life be part of a palliative approach? yes/no

**Questions on the decision-making process**

21. Is the introduction/withdrawal/withdrawal of artificial nutrition for Mrs C. discussed interprofessionally in your practice? yes/no

22. With which professional do you share the discussion?

- Medical colleagues? yes/no

- Nursing colleagues? yes/no

- Care assistant colleagues? yes/no

- Other professionals? yes/no

23. Is the introduction/stopping/withdrawal of artificial nutrition in Mrs C.'s case a subject to be discussed?

- Only with Mrs C.?yes/no

- Only with her family? yes/no

- With both? yes/no

24. If Mrs C. is incapable of discernment or cannot communicate, who do you talk to?

- Relatives

- Interprofessional team

- GP

- None of the above

25. In your opinion, how important is the opinion of the doctor in charge in the decision to stop/withdraw artificial nutrition at the end of life? Little/moderate/decisive?

26. In your opinion, how important is the opinion of the nurse in the decision to stop/withdraw artificial nutrition at the end of life? Little/moderate/decisive?

27. In your opinion, how important is the patient's opinion in the decision to stop/withdraw artificial nutrition at the end of life? Little/moderate/decisive?

28. In your opinion, how important is the opinion of relatives in the decision to stop/withdraw artificial nutrition at the end of life? Little/moderate/decisive?

29. What decision-making criteria do you use when starting/stopping/withdrawing artificial nutrition?

- 1- Patient's life expectancy? yes/no

- 2- Patient's quality of life? yes/no

- 3- Patient's nutritional status? yes/no

- 4- Patient's age? yes/no

- 5- Comorbidities? yes/no

30. In relation to question 29, rank the importance of these criteria

- 1- Patient's life expectancy? low/moderate/high

- 2- Patient's quality of life? low/moderate/high

- 3- Patient's nutritional status? low/moderate/strong

- 4- Patient's age? low/moderate/strong

- 5- Comorbidities? low/moderate/strong

31. In the case of Mrs C., what criteria would prompt you to start artificial nutrition?

32. In the case of Mrs C., what criteria would lead you to abandon artificial nutrition?

33. Which ethical principle influenced your decision to stop artificial nutrition?

- The principle of justice? yes/no

- The principle of beneficence? yes/no

- The principle of non-maleficence? yes/no

- The principle of autonomy? yes/no

34. In connection with question 33, rank the importance of these criteria

- The principle of justice?

- The principle of beneficence?

- The principle of non-maleficence?

- The principle of autonomy?

35. If you were in Mrs C's situation, would you be for or against the use of artificial nutrition? for/against

36. If you were to lose your capacity for discernment, would you still be for or against?

37. What level of certainty would you have if you had to take the decision to start artificial nutrition for Mrs C.? Low/moderate/strong

38. What level of certainty would you have if you had to make a decision not to start artificial nutrition for Mrs C.? Low/moderate/strong

39. What would you do if Mrs C. did not agree with your decision?

- Would you accept her opinion? yes/no

- Disregard her opinion? yes/no

- Organise a meeting with her family? yes/no

- Organise an interprofessional meeting? yes/no

- Ask the ethics council for advice? yes/no

40. If your team disagrees on a decision, what do you do?

- Follow the majority? yes/no

- Follow the patient's advice? yes/no

- Do you follow the opinion of relatives? yes/no

- Do you consult the ethics council? yes/no

41. After starting artificial nutrition, do you plan to reassess its use? yes/no

42. Was the need to reassess artificial nutrition discussed with your patient before it was started? yes/no

43. And with your patient's family? yes/no

44. With whom do you carry out the reassessment? :

- Patient yes/no

- Relatives yes/no

- Physician colleagues yes/no

- Nursing colleagues yes/no

- Dietician yes/no

45. What criteria do you use to reassess artificial nutrition? (free text)
